# A study of flotation-REST (restricted environmental stimulation therapy) as an insomnia treatment

**DOI:** 10.5935/1984-0063.20210012

**Published:** 2022

**Authors:** Annika Norell-Clarke, Kristoffer Jonsson, Annecharlotte Blomquist, Rolf Ahlzén, Anette Kjellgren

**Affiliations:** 1 Karlstad University, Department of Social and Psychological Studies - Karlstad - Värmland - Sweden.; 2 Karlstad University, Centre for Research on Child and Adolescent Mental Health - Karlstad - Värmland - Sweden.

**Keywords:** Flotation Therapy, Insomnia, Multiple Baseline, Relaxation, Sensory Deprivation, Sleep

## Abstract

**Objectives:**

Flotation-REST is a treatment for deep relaxation, where a person is contained in a stimuli-restricted environment and floats in water with high salt content. The aim was to investigate the effects from flotation-REST on people with insomnia diagnosis, as previous studies of flotation-REST have demonstrated some effects on sleep but have limitations regarding sample selections and sleep measures.

**Material and Methods:**

Six participants were recruited through an outpatient psychiatry clinic and posters on a university campus. All participants fulfilled criteria for insomnia diagnosis and four fulfilled criteria for major depressive disorder. Using a single case experimental design, daily changes were investigated on sleep logs regarding sleep onset latency (SOL), wake after sleep onset (WASO), total sleep time (TST), and sleep efficiency over the course of 12 sessions consisting of 45 min of flotation-REST. No other treatments were offered simultaneously. Questionnaire data on insomnia severity (the ISI) and depressive severity (the MADRS) were also collected.

**Results:**

Three participants improved on their most salient insomnia symptom (long SOL or WASO), and two improved on sleep efficiency. The improvements were maintained 2 months after treatment. Insomnia severity decreased for three patients, whereas depressive severity decreased for five. No changes in TST were found and two patients did not improve on any sleep measure. The two participants who benefitted the most were students in their 20s.

**Discussion:**

The results were mixed. Flotation-REST may be beneficial for young adults with sleep-onset insomnia but more research is warranted.

## INTRODUCTION

Insomnia is a common health complaint that affects about 10 % of the general population and means difficulties falling asleep, falling back to sleep, or waking up too early in the morning^1^. For a diagnosis, the nighttime symptoms must also affect the daytime in that sufferers report a decreased ability to function or that they are worried about their sleep. Evidence-based treatment options for insomnia include cognitive behavioral therapy (CBT-I) and pharmacological treatments^2^ but many people with insomnia use methods within the complementary and alternative medicine (CAM) umbrella such as herbal medicine or acupuncture, alone or in combination with other insomnia treatments^3,4^. Although many CAM therapies for insomnia have been evaluated^5^, there are still methods whose effects on sleep are unknown, such as flotation-REST.

Flotation-REST (restricted environmental stimulation therapy) means floating comfortable on the back inside of a dark and quiet isolation tank, filled with salt saturated water that is maintained at about 35 degrees Celsius. The high salt content in the water gives high buoyancy, which makes it possible to float on top of the water. By keeping the water at skin-temperature, sensory input to the somatosensory system is reduced. Earplugs are used to further minimize auditory stimuli. Modern research on flotation-REST has established a treatment protocol consisting of 12 flotation sessions (each of 45 minutes) over a 7-week period, and this has been suggested as sufficient to reach therapeutic effect when used as an intervention for chronic pain conditions as well as stress related ailments^6^. Contemporary research on flotation-REST has indicated this method as a promising treatment for chronic pain conditions^7,8^, stress-related ailments^9,10^, generalized anxiety disorder^11^, as well as a preventive health care intervention^12^. In addition, a meta-analysis has indicated flotation-REST primarily as an effective method for stress reduction, increasing well-being, and performance enhancement^13^. Flotation-REST is often advertised as treatment of fatigue, depression and stress - health problems commonly comorbid with sleep problems - and sometimes promoted as an insomnia treatment^14^ but the effects from flotation-REST on insomnia have not been sufficiently investigated.

A recent systematic review concluded that flotation-REST might be a promising treatment for sleep problems^15^. For example, studies have reported increased post-treatment sleep quality for healthy workers^12^ (a non-psychometrically tested scale with 11 items), people with stress problems^9^ (“rate your sleep quality from 0-100%”), people with burnout syndrome^16^ (a non-psychometrically tested scale with 11 items), and people with generalized anxiety disorder^11^ (the Pittsburgh sleep quality index). People with chronic pain reported shorter sleep onset latency, but no changes in sleep duration after therapy in one study^8^. Only one previous study has investigated the effects from flotation-REST on people with primary insomnia. No immediate post-treatment effects on sleep onset latency (SOL) or total sleep time (TST) were reported but there were some improvements in SOL 12 weeks later (sleep diary and a nightly voice-activated recording device)^17^.

In the above mentioned studies, sleep has been measured with varying psychometrical quality but no study to date has investigated the effects from flotation-REST on people diagnosed with modern insomnia criteria, or combined sleep diaries with psychometrically sound questionnaires to measure sleep. In addition, the sole reliance on questionnaires to measure effects on sleep limits the conclusions that can be drawn as individuals’ general reports of their sleep sometimes differ from the night by night information sleep diaries provide^18^. The only previous study that investigated people with primary insomnia and used sleep diaries excluded anyone with signs of comorbid medical, psychological or behavioral problems and only included those who endorsed sleep onset problems^17^, whereas a modern definition of insomnia diagnosis allows for comorbidity and for sleep problems that manifest later than the first sleep onset, as well as requiring daytime suffering and a specified duration of sleep problems^1^. The use of a recording device that signaled every ten minutes after bedtime may also have disrupted the participants’ sleep. Hence, the results from this study may have been dampened. Therefore, whether or not people with insomnia can attain improved sleep from flotation-REST is not fully answered by the previous literature.

When treatments are tested in novel contexts, a small scale study, focusing on a few people carefully may be a wise first step to save resources^19^. In single case experimental design (SCED), individuals serve as their own control, as repeated measures during a baseline period are compared to repeated measures during an intervention period^19^. If data regard the measure of interest during the baseline are stable (or deteriorating), an assumption is made that this development would continue over time. Positive changes that only occur after treatment has started, or become more pronounced, are attributed to the treatment. An advantage with SCEDs is that it is possible to get comprehensive data on change processes for a few individuals with relatively little investment, which makes these designs suitable when novel treatments are tested and the hypotheses regarding whether or not an intervention has an effect.^19^. Replication strengthens the conclusions that can be drawn and this can be achieved with multiple participants and/or multiple measures within the same study. Randomization to different starting times for the intervention controls for some validity threats such as history.

The aim of the current study was thus to investigate the effects of flotation-REST on insomnia severity and nighttime symptoms (sleep onset latency, wake after sleep onset, total sleep time, and sleep efficiency) by using a multiple single case experimental design. Our main hypothesis, in line with flotation previous research on sleep quality, was that general subjective suffering from insomnia would decrease over the course of treatment. We also expected sleep onset latency and wake after sleep onset to improve, as this was plausible from a relaxation perspective. We did not expect total sleep time (TST) or sleep efficiency (SE) to change as the effects from other insomnia treatments on TST are at best modest^20,21^ and changes in SE are associated with instructions to change sleep habits, specifically spending less time in bed^22^.

## MATERIAL AND METHODS

A multiple baseline single case experimental design was used to test the effects of 12 sessions over 7 weeks of flotation-rest on 6 participants with insomnia. A follow-up measure was conducted two months after therapy. Prior to recruitment, the study protocol was approved by the Regional Ethics Vetting Board in Uppsala, Sweden (No.: 2015/303). Written informed consent was obtained from the participants. The clinical trial was registered retrospectively at http://www.ANZCTR.org.au/ACTRN12617001153303.aspx For demographic and clinical characteristics of the participants, see Table 1. When designing the study, care was taken to closely follow general quality criteria of SCEDs, for example (but not limited to) sufficiently defined outcome criteria, a long enough baseline period, randomization of treatment start times, a minimum of five observations in each study phase for main outcome variables (baseline, treatment, and follow-up), and inter-subject replication (multiple participants)^23,24^. Due to assumed irreversible effects of the therapy, intra-subject replication (varying baseline with treatment phases several times) was not conducted.

**Table 1:** Demographic descriptions of the participants and results on insomnia and depressive severity.

Participant no.	1	2	3	4	5	6
**Demographics**						
Gender	Woman	Man	Woman	Woman	Woman	Woman
Age	20-29	20-29	20-29	30-39	50-59	40-49
In a romantic relationship	Yes	No	No	Yes	Yes	Yes
Born in Sweden	Yes	Yes	Yes	Yes	Yes	Yes
Completed education	Grammar school	Upper secondary school	Upper secondary school	Upper secondary school	University	Upper secondary school
Occupational status	Student	Student	Sick leave	Employed	Employed	Sick leave
Recruitment	Campus poster	Campus poster	Psychiatrist	Campus poster	Campus poster	Psychiatrist
**Insomnia and depressive symptoms**						
Insomnia symptoms	Difficulties initiating sleep	Difficulties initiating sleep	Difficulties initiating & maintaining sleep	Difficulties initiating & maintaining sleep	Difficulties initiating sleep	Difficulties maintaining sleep
Insomnia duration (Y)	10	3	15	16	16	20
Depressive duration (Y)	3	1	1	17	N/A	N/A
Insomnia severity at baseline (ISI)	17 (Moderate)	13 (Moderate)	17 (Moderate)	23 (Severe)	15 (Moderate)	21 (Moderate)
Insomnia severity after treatment	8 (Sub-threshold)	0 (Sub-threshold)	23 (Severe)	24 (Severe)	11 (Moderate)	4 (Sub-threshold)
Insomnia severity at follow-up	13 (Moderate)	4 (Sub-threshold)	23 (Severe)	N/A	15 (Moderate)	3 (Sub-threshold)
Depressive severity at baseline (MADRS-S)	20 (Moderate)	12 (Mild)	12 (Mild)	26 (Moderate)	10 (Mild)	14 (Mild)
Depressive severity after treatment	8 (Mild)	7 (Mild)	10 (Mild)	14 (Mild)	5 (Sub-threshold)	2 (Sub-threshold)
Depressive severity at Follow-up	9 (Mild)	4 (Sub-threshold)	16 (Mild)	N/A	14 (Mild)	10 (Mild)

Notes: ISI = The insomnia severity index; MADRS-S = The Montgomery Åsberg depression rating scale self-rated; Y = Years; ISI cutoffs: ≥8 = subthreshold insomnia, ≥15 = moderate insomnia, and ≥22 = severe insomnia; MADRS-S cutoffs: ≥7 = mild depression, ≥20 = moderate depression, and ≥35 = severe depression.

### Procedure

Participants were recruited via posters on campus and through a psychiatrist at an outpatient psychiatric clinic (RA). Those who were interested in the study contacted the project group at the university and went through a short initial telephone screening (KJ). The second part of the screening took place at the university and included structured clinical interviews with the Duke structured interview for sleep disorders^25^ (DSISD) and the structured clinical interview for DSM-IV axis disorders^26^ (SCID-I) by a licensed clinical psychologist (ANC). For the last step of the screening, the participants were asked to complete sleep diaries for two weeks.

The inclusion criteria were: 1) insomnia diagnosis (DSISD); 2) SOL or WASO exceeding 30 min three nights per week (sleep diaries); 3) age between 18-65; 4) not currently receiving other psychological treatments for insomnia; 5) on a stable dose of medication over the last three months (if receiving pharmacological treatment). The exclusion criteria were: 1) sleep-disturbing medical conditions, medications or untreated sleep disorders that may explain insomnia symptoms (DSISD); 2) other health conditions that would make flotation-rest unsuitable (e.g., pacemaker, open wounds); 3) sleep-disturbing mental problems (SCID-I) that may be worsened during the study period (e.g., recent manic episode); 4) suicide ideation or suicidal behaviors (SCID-I).

Those who fulfilled all criteria over the three steps of the screening process were offered to participate in the study (n=6). Those who fulfilled all criteria with the exception of the sleep pattern criteria were invited to test the flotation-REST but their results were not measured (n=2). Participants were randomized to different start dates for the flotation-REST between March and August, 2016. The randomization was conducted by a study independent researcher who drew paper “lottery tickets” with various start dates. Where accommodation was needed to fit participants’ menstrual cycle, the treatment start was adjusted accordingly so that the fourth week of treatment would be treatment-free.

### Measures

Sleep onset latency, wake after sleep onset, total sleep time, and sleep efficiency were measured with sleep diaries. Subjective insomnia suffering was measured with the insomnia severity index^27^ (ISI), which is commonly used in insomnia treatment research and has sound psychometric properties^28^. Scores of 8-14 indicate subthreshold insomnia, 15-21 moderate insomnia, and 22-28 severe insomnia^27^, although it should be noted that scoring ≥10 is a psychometrically established cutoff for detecting insomnia in the general population^28^. The Montgomery Åsberg depression rating scale^29^ (MADRS-S) measures depression and has sound psychometric properties^30^. Scores of 7-19 indicate mild depression, 20-34 moderate depression, and 35-60 mark severe depression^31^. The MADRS-S was used as a proxy for daytime symptomatology. The anxiety and preoccupation about sleep questionnaire^32^ (APSQ) and the pre-sleep arousal scale^33^ (PSAS) were used as measures of mental and somatic arousal, respectively.

### Flotation-REST

Float tanks (270cm x 150cm x 130cm) situated in quiet rooms were used. The float tanks contained water (0.3m in depth) held at skin temperature (35 degrees Celsius) that were saturated with Epsom salt (magnesium sulphate). The float tanks were insulated to minimize visual and auditory stimuli, and earplugs were used during the float sessions. Toilets and showers could be accessed in the float rooms and the float rooms could be locked from inside by the participants. Staff had access to the room and were present in an adjoining room during all sessions and could be called upon by an alarm button located inside the float tanks (no such incidents were reported during the study). Prior to the first flotation sessions, the participants were instructed to keep the light out during the float session, and to shower before and after each session. The treatment consisted of 12 sessions (each of 45 minutes) of flotation-REST over a seven-week period, with two sessions a week. The fourth week was treatment free, in order to simulate the break needed by menstruating participants. The flotation sessions (bookings, instructions and contact with the participants before and after each session) were managed by AB.

### Analyses

First, daily measures of sleep onset latency, wake after sleep onset, total sleep time, and sleep efficiency were displayed in graphs to facilitate visual inspection of changes over baseline, treatment and the follow-up period. Visual inspection is the core method of displaying SCED results, which is sometimes complemented by statistics that are suitable for single case data^19,34^. In the current study, non-overlap of all pairs (NAP) were calculated. Techniques to determine the extent of non-overlapping data between different study phases have been important methodological tools in single case research for over 30 years. To put it simply, the greater the difference between phases, the stronger the effect of the intervention. A comparison of the leading non-overlap techniques has yielded that the non-overlap of all pairs overcomes the shortcomings of the previous techniques^35^, thus it was chosen for the current study. In short, calculations of non-overlap means comparing each data point in one phase with each point of another phase to determine, as in our study, whether the specific sleep onset latency for one night of the baseline period was longer than during a specific night during the treatment. The number of non-overlapping comparisons are then divided by the total amount of comparisons to calculate the percentage of non-overlap. The calculations were conducted with the NAP Calc software by IMC Soft^36^. Percentages between 93-100% indicate a strong effect of treatment, 66-92% a medium effect, and below 65% a weak or questionable effect^35^.

## RESULTS

### Nightly sleep diary data

**Figure 2:**
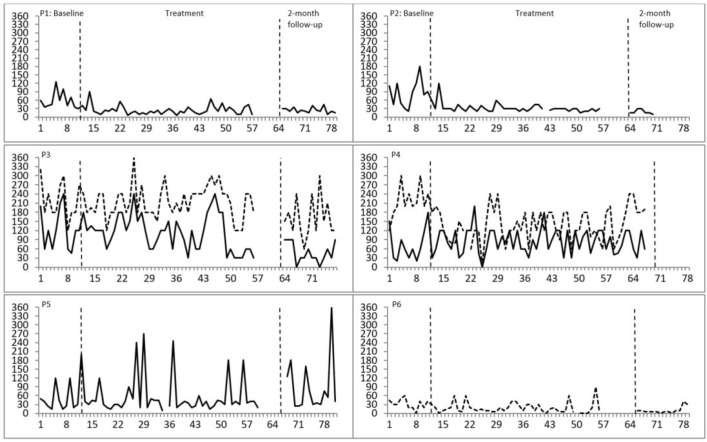
Nightly insomnia symptoms: Sleep Onset Latency (SOL) and Wake After Sleep Onset (WASO). P = Participant; Solid line = Sleep onset latency in minutes; Dotted line = Time awake after first sleep onset in minutes. Only sleep issues relevant for the specific participant's insomnia symptomatology are displayed (as supported by sleep diaries and their own descriptions). Percentages of nonoverlapping data between baseline and treatment for each participant: P1: SOL = 89.70%; P2: SOL = 82.06%; P3: SOL = 2.44%, WASO = 40.48%; P4: SOL = 32:07%, WASO = 81.46%; P5: 45.24%; P6: WASO = 65.68%. Percentages between 90-100% indicate a high effect of treatment, 70-89% a moderate effect, and below 70% a questionable or no effect.

*Sleep onset latency*: during baseline, participants 1, 2, 3, 4, and 5 reported sleep onset latencies exceeding 30 min three times or more per week. During treatment, moderate improvements in sleep onset latency were observed for participant 1 and 2 (Figure 2).

*Wake after sleep onset*: three participants (3, 4, and 6) reported wake after sleep onset issues exceeding 30 min three times or more per week during baseline. A moderate improvement in wake after sleep onset was observed for participant 4 (Figure 2).

**Figure 3:**
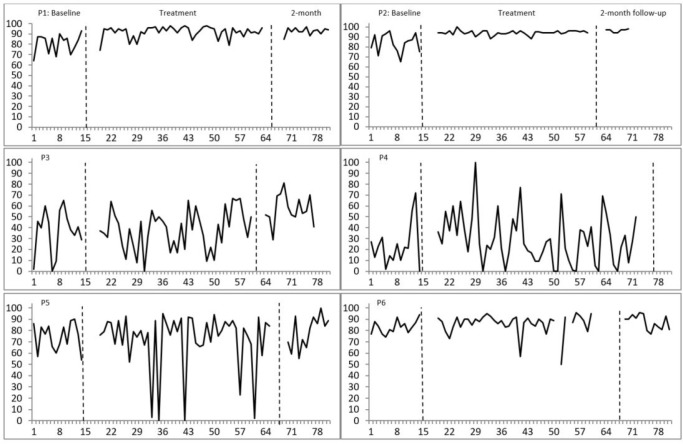
Sleep efficiency in percentage per night. P = Participant. Higher numbers indicate greater percentages of time asleep in bed out of the total time spent in bed. Percentages of nonoverlapping data between baseline and treatment for each participant: P1: 87.46%; P2: 84.50%; P3: 49.49%; P4: 43.38%; P5: 55.93%; P6: 68.21%. Percentages between 90-100% indicate a high effect of treatment, 70-89% a moderate effect, and below 70% a questionable or no effect.

*Sleep efficiency*: moderate improvements on sleep efficiency were also observed for participants 1 and 2 (Figure 3).

*Total sleep time*: there were no reliable changes in total sleep time for any participant (data available on request).

All the above results were supported by NAP calculations (Figures 2 and 3). To conclude, two out of six participants improved on more than one sleep parameter, one participant on one parameter whereas the sleep of three participants did not change over treatment.

### Questionnaire data on insomnia severity and depressive symptoms

Before treatment, all participants reported insomnia severity at a moderate or severe level on the ISI (see Table 1 for data on the ISI and the MADRS). After treatment, participants 1, 2, and 6 reported subthreshold insomnia severity. The insomnia severity for participant 3 had worsened whereas the severity level for participant 4 and 5 was unchanged. Regarding depressive symptoms, as measured by the MADRS, all participants except participant 3 reported distinctively lower levels of depressive severity after treatment. The results from the APSQ and PSAS are available in Figure 4 in the supplementary material.

**Figure 4:**
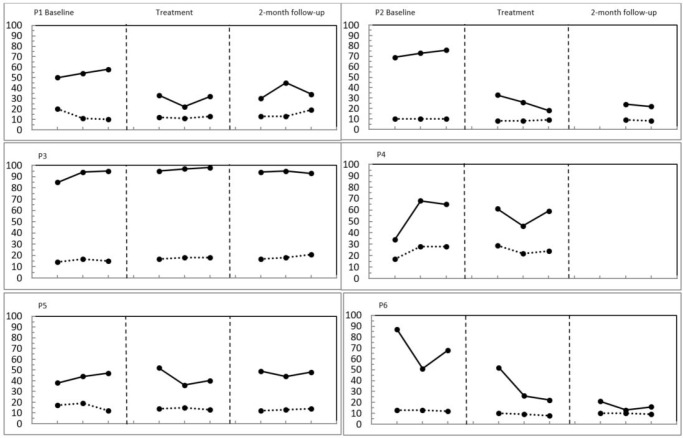
Sleep-related worry and pre-sleep arousal across baseline, treatment and follow-up. P = Participant; Solid line = Anxiety and preoccupation about Sleep Questionnaire (worry); Dotted line = Pre-Sleep Arousal Scale.

## DISCUSSION

The aim of the study was to investigate the effects from flotation-REST on people with insomnia diagnosis by using a repeated single case experimental design. Our hypothesized improvements in subjective suffering from insomnia and the time to fall asleep or fall back to sleep were observed in three participants respectively (1, 2, and 6; 1, 2, and 4). Those who reported improved sleep also reported lower degrees of depressive severity over time, which is in line with previous research which have demonstrated that treatments for insomnia also have an effect on depression^37^. However, some participants did not report any beneficial effects from flotation-REST. The mixed results can be understood based on what the mechanisms in flotation-REST may be and the characteristics of the specific participants.

Starting with the proposed mechanisms, lowered arousal has been reported as a treatment effect from flotation^13^. Further, treatments that promotes lower arousal, namely relaxation techniques, have some effect on both depression and insomnia^38,39^, which might explain the results in these two domains in the current study. As heightened mental and physiological arousal is one of the maintaining factors of insomnia, improved sleep should co-occur with less arousal. Looking at the reported sleep-related worry and arousal, participants 1, 2, and 6 demonstrated less worry after the treatment started. This was not obvious for self-reported physical arousal. It is possible that those who experienced less mental arousal in conjunction with treatment also benefited from better sleep. However, improved sleep may have led to less worry. Future research should therefore investigate objective measures of arousal and establish a time line regarding which effect comes first (it should be noted that we did not choose particularly tense people, people with high sleep-related arousal or those who might specifically benefit from some kind of relaxing intervention).

Although our sample is too small to draw any reliable conclusions regarding the demographics of our participants in relation to the outcome, there are a few observations that might offer ideas for future research on whose insomnia that flotation-REST might be helpful for. First, the two participants who reported the greatest improvements on the sleep parameters (sleep latency, sleep efficiency, and insomnia severity) were both fulltime university students in their 20s with sleep onset issues as their primary insomnia concern. Second, the two participants on sick leave, who had been recruited through psychiatry, did not report any improvement on the daily measures of sleep. From the screening process, we know that there were comorbid problems beyond depressive symptomatology (not reported for confidentiality reasons). It is therefore difficult to know how youth, sleep symptomatology, or comorbid health issues might have influenced the participants’ insomnia or the potential to benefit from flotation-REST regarding their sleep. Future studies should investigate potentially moderating factors of flotation-REST - such as age, occupational status, and comorbidities - to illuminate this matter.

Surprisingly, the sleep efficiency improved for two participants, despite no instructions to change their sleep habits. The participants were not instructed to change their sleep habits in any way, or given any information about sleep that might have had a normalizing or calming effect (as in the psychoeducation aspect of CBT for insomnia) but some of the effect can be explained by spending less time awake in bed as a result from falling asleep faster. Regarding CBT-I, we are curious about the effects of CBT combined with flotation-REST, and are currently planning a study, which will test the effects on insomnia.

The study design has some strengths and limitations that should be considered. First, the mixed results make it difficult to conclude whether or not flotation-REST has an impact on insomnia symptoms. Although every participant should be considered as an experiment in single case research^40^, replication over participants is expected to confirm the effects. Second, events or treatments outside the study could have had an impact on the results. The participants were asked to report if they started any new treatment or experienced life events that might impact their sleep. No such incidents were reported.

To conclude, there is little research conducted on the effects from flotation-REST on insomnia, despite commercial claims that flotation is beneficial for sleep. This study contributes to the knowledge by testing the therapy on people with diagnosed insomnia and by investigating sleep with gold standard insomnia measures.

## Figures and Tables

**Figure 1:**
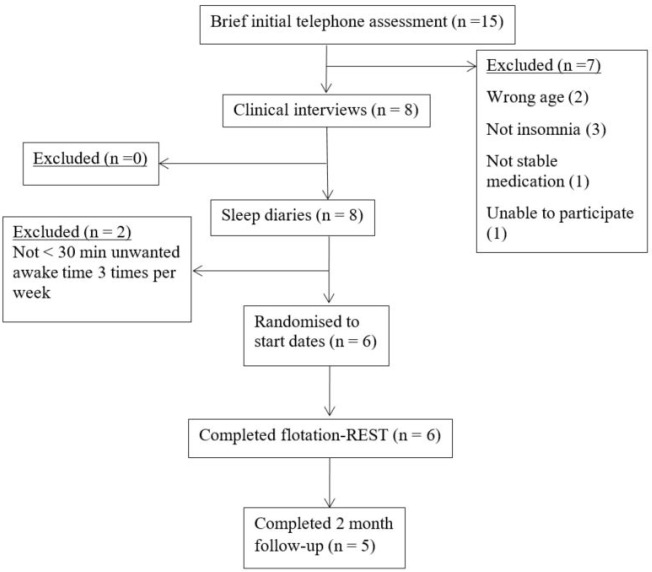
Participant flow chart from recruitment to follow-up. Participant flow chart from recruitment to follow-up.
